# The role of the Mediator complex in fungal pathogenesis and response to antifungal agents

**DOI:** 10.1042/EBC20220238

**Published:** 2023-09-13

**Authors:** James O'Connor-Moneley, Leenah Alaalm, Gary P. Moran, Derek J. Sullivan

**Affiliations:** Microbiology Research Unit, Division of Oral Biosciences, Dublin Dental University Hospital, University of Dublin, Trinity College Dublin, Dublin, Ireland

**Keywords:** Drug resistance, Fungi, Mediator, Pathogenic, Virulence, Yeast

## Abstract

Mediator is a complex of polypeptides that plays a central role in the recruitment of RNA polymerase II to promoters and subsequent transcriptional activation in eukaryotic organisms. Studies have now shown that Mediator has a role in regulating expression of genes implicated in virulence and antifungal drug resistance in pathogenic fungi. The roles of specific Mediator subunits have been investigated in several species of pathogenic fungi, particularly in the most pathogenic yeast *Candida albicans*. Uniquely, pathogenic yeast also present several interesting examples of divergence in Mediator structure and function, most notably in *C. glabrata,* which possesses two orthologues of Med15, and in *C. albicans,* which has a massively expanded family of Med2 orthologues known as the *TLO* gene family. This review highlights specific examples of recent progress in characterizing the role of Mediator in pathogenic fungi.

## Introduction

Fungi are an important component of the biosphere and exist in a wide variety of anatomic niches as a minor component of the human microbiome. Under certain circumstances, a range of yeast and filamentous fungal species can cause infections and are responsible for significant levels of morbidity and mortality in humans, with up to 1.5 million people dying from fungal infections each year [1,2]. Compared with bacterial pathogens the study of fungal involvement in human disease has been largely neglected [3]. In addition, development of antifungal agents to treat fungal infections is hampered by the fact that they are eukaryotes and therefore share many traits in common with their human hosts.

While a growing number of filamentous fungi are responsible for opportunistic infections [4], the most common cause of serious fungal diseases are yeast species belonging to the genus *Candida*. One species in particular is widely recognized as one of the most important human fungal pathogens. For the most part, this species, *Candida albicans*, exists as a commensal member of the normal microbiota in the oral cavity, the gastrointestinal tract and the vagina of humans [4–6]. Although it is mainly kept in check by the host immune response, if there is an imbalance, due to changes within the mucosal microbiota and/or a defective host immunity, *C. albicans* can overgrow and cause a wide range of superficial and systemic infections in at risk individuals [4,5,7]. The genus *Candida* comprises many species, and although *C. albicans* is usually the most frequently encountered and best studied, in recent decades the prevalence of non-*albicans Candida* species, such as *C. glabrata* and *C. parapsilosis*, has increased [4,7]. In addition, a new species, *Candida auris* has recently emerged as a pathogen of concern, primarily due to its enhanced resistance to antifungal drugs [8]. Given the commensal nature of the relationship between *Candida* species and their human hosts, the pathogenesis of candidal infections is complex and involves the interplay between the host immune response and a range of putative virulence factors [6,7]. These include the expression of gene families that encode adhesins and proteinases, the ability to form biofilms, to transition reversibly between yeast and filamentous (i.e., hyphal) forms and, in the case of *C. albicans*, the ability to produce the recently discovered cytotoxin candidalysin (Ece1) [6,9]. One of the reasons *C. albicans* is considered to be among the most successful fungal pathogens in humans is its ability to colonize and infect a diverse range of host anatomic sites [9,10]. This adaptability is likely facilitated by the yeast rapidly sensing changes in the surrounding environment through a complex network of signal transduction pathways [11] and chromatin modification [12], which can influence transcription and therefore gene expression.

## Mediator complex in yeast: structure and function

**Figure 1:**
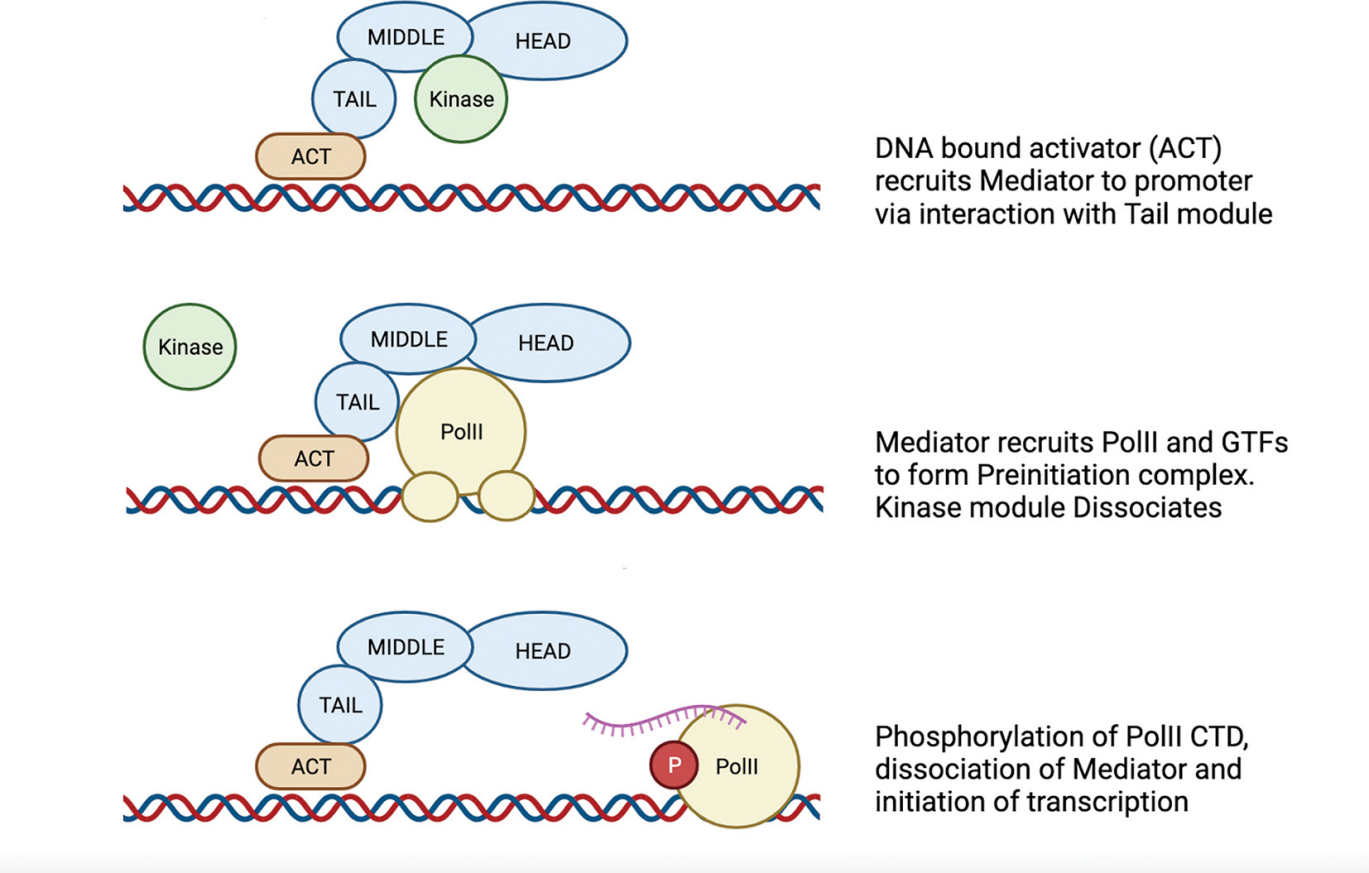
The role of Mediator in transcription A diagram (modified from Petrenko et al. [60]) summarizing the basic role of Mediator in transcription. Mediator is recruited by DNA-bound transcription factors to the promoters of genes via interactions with the tail module (Med2,3,5,15,16). Recruitment of Pol II and general transcription factors (GTFs) occurs following dissociation of the regulatory kinase module. Phosphorylation of the C-terminal domain (CTD) by C-terminal domain kinases such as Kin28 then releases Pol II from Mediator and allows mRNA transcription to commence. This figure was generated using Biorender (https://biorender.com/).

The transcription of protein encoding genes into mRNA involves a complex interplay between specific DNA sequences upstream of the open reading frame to be expressed (the promoter) and a range of proteins, including, in eukaryotes, the main protagonist, RNA polymerase II (Pol II). One important and critical player in the transcription of many of the genes that are required for virulence and host adaptation is the Mediator complex, which is a conserved and essential multi-subunit complex of polypeptides [13–16]. Our current understanding is that Mediator acts as an intermediary between regulatory sequences and DNA-bound activators or repressors, to link or to interfere, respectively, with the basal transcriptional machinery in all eukaryotes. Mediator interacts with other co-activator complexes to help in the formation of the preinitiation complex (PIC) in the case of transcriptional activation (Figure 1). In addition, it can also prevent this interaction, leading to transcriptional repression [13–16],[60].

The structure and function of the Mediator complex has been well studied in the model yeast species *Saccharomyces cerevisiae*. In this species, the Mediator complex consists of 25 individual subunits that are distributed between four distinct modules: seven subunits comprise the head module (Med6, Med8, Med11, Med17, Med18, Med20 and Med22), nine subunits comprise the middle module (Med1, Med4, Med7, Med9, Med10, Med14, Med19, Med21 and Med31), five subunits comprise the tail module (Med3, Med5, Med15, Med16 and Med2), and finally four subunits comprise the kinase module (Cdk8, CycC, Med12 and Med13) [13–16]. The kinase module can function independently from Mediator, but when it is associated with Mediator it prevents association with RNA Pol II [16]. The kinase module can also modify the activity of some Mediator subunits by post-translational modification (e.g., phosphorylation) [15,17]. In fungi, it has been reported that approximately half of Mediator subunits are essential, and these have approximately 20–30% amino acid identity with their counterpart subunits in mammals [18–21]. With the exception of *S. cerevisiae*, the role of Mediator in fungal biology is relatively poorly understood, particularly in medically important fungal species.

## The role of Mediator in fungal pathogenesis

Despite the central role played by Mediator in controlling fungal gene expression, how the complex influences the expression of genes involved in host–pathogen interactions has been poorly investigated. In the human pathogenic yeast species, *Cryptococcus neoformans*, it has been shown that Ssn8, a C-type cyclin and a component of the Mediator kinase module is required for virulence and cell wall integrity. The present study also showed that Ssn8 acts as a negative regulator in the mating process and is involved in the suppression of invasive growth, formation of the capsule and melanin production [22]. Another member of the kinase module, Ssn801 was also identified and shown to be also required for virulence and to regulate responses to oxidative stress [23].

In *C. albicans*, Uwamahoro et al. [24] showed that representative subunits from specific modules of the Mediator complex, such as Med31 from the middle module, Med20 from the head module, and Med13/Srb9 from the kinase module play key roles in virulence-associated attributes, including the ability to form hyphae and biofilms. Deletion of *MED31* was shown to reduce the expression of adhesin-encoding *ALS* genes and to reduce the virulence of *C. albicans* in a nematode worm infection model. Interestingly, while Med13/Srb9 and Med31 appear to play a role in activating *ALS* genes in *C. albicans*, in *S. cerevisiae* they appear to act as repressors of the *FLO* genes, a family that encodes proteins involved in cell–surface and cell–cell interactions [25]. This suggests that although there is a high degree of conservation between *Saccharomyces* and *Candida* Mediator subunits, they can regulate different aspects of virulence in a species-specific manner [24].

Deletion of the gene encoding the Med7 component of the middle module of *C. albicans* Mediator (which has been shown to be non-essential in *C. albicans* but essential in *S. cerevisiae*) affected the mutant’s ability to metabolize non-traditional carbon sources (e.g., galactose and fructose), to form biofilms, to transition from yeast to hyphae, and to colonize the mouse gastrointestinal tract [26]. Chromatin immunoprecipitation (ChIP) analysis revealed that Med7 was found to associate not only with the promoter regions but also with intergenic and coding regions of target genes (both active and inactive), including many that have been suggested to be associated with virulence in this fungus. These data combined with transcription profiling data suggest that Med7 appears to be specifically involved in the control of expression of the glycolytic pathway and the fungal gene regulator (*FGR*) family of genes associated with hyphal growth. The ChIP data also suggest that Med7 shows higher binding levels in hyphal cells, correlating with increased transcriptional activity [26]. Med7 was also found to bind to the *WOR1* and *WOR2* genes [26] suggesting a possible role in white-opaque epigenetic switching in *C. albicans*. Data from further Mediator subunit-encoding gene deletion studies suggest that different complex components differentially regulate white-opaque switching in *MTL* homozygous strains. For example, deletion of *MED12*, *MED3* and *MED15* negatively regulates the switch from white to opaque, while deletion of *MED20* and *MED1* have an opposite effect as they were found to positively regulate the switch from white to opaque. On the other hand, deletion of *MED9* and *MED16* increased the rate of switching in both directions. However, deletion of *MED5* had no detectable effect on switching [27].

Deletion of the gene encoding the tail module component Med15 in the emerging opportunistic yeast pathogen *Candida lusitaniae* resulted in defective cell separation, pseudohypa formation and mating. Proteomic analysis revealed that this mutation led to the up-regulation of genes involved in stress responses and utilization of alternative carbon sources. Indirect data from this study also suggest that Med15 may contribute to, but is not essential for, wild-type levels of virulence [28]. Screening a mutant library of the filamentous fungal pathogen *Aspergillus fumigatus* revealed that down-regulation of a gene, *AfMED15*, encoding a protein with a fungal Med15 domain, affected a wide range of phenotypes, including conidiation and virulence in the *Galleria mellonella* wax moth infection model. Interestingly, over-expression of this gene was lethal for the fungus, possibly due to altered transport of divalent cations, such as calcium and iron [29].

## The role of Mediator in the response of fungi to antifungal drugs

The range of drugs available to treat fungal infections is small when compared with the numbers of anti-bacterial agents that are currently available [30]. Exposure of pathogenic fungi to antifungal agents has led to the emergence of antifungal drug resistance, caused by a range of resistance mechanisms, including induced changes in drug binding affinity, reduced expression of drug targets, formation of protective biofilms and increased drug efflux pump gene expression [31].

The potential involvement of Mediator in the development of antifungal resistance has been of increased interest in recent years and has been studied in several fungal species, including *Candida* species and the filamentous *A. fumigatus* [32–35]. Of these mechanisms, the involvement of Mediator in the regulation of the transcription factor Pdr1, responsible for controlling efflux pump gene expression, is the most comprehensively described across several fungal species [32–40]. Among the first studies to implicate Mediator involvement in antifungal drug resistance were studies in *S. cerevisiae*, which showed ScPdr1/3-dependant recruitment of Mediator to the drug responsive element (DRE) in the promoter of the ABC-transporter-encoding gene *ScPDR5*, in response to gain of function mutations, induction by azole and loss of the mitochondrial genome [36,41–43]. Induction of ScPdr5 efflux pump expression resulted in resistance to ketoconazole which was shown to be dependent on the ScMed15 component of the tail module of Mediator [36]. This regulatory mechanism was mirrored in the related fungal pathogen *C. glabrata*. Interactions between the ScMed15 ortholog, CgMed15A, and the transcription factor CgPdr1 directly affected the expression of the ABC transporter encoding genes *CgCDR1* and *CgCDR2* (orthologs of ScPDR5) [36,42]. The up-regulation of these transporters, especially CgCdr1, leads to increases in azole resistance [37]. Interestingly, the second *MED15* ortholog in *C. glabrata, CgMED15B*, had no observed effect on ABC transporter expression or azole resistance [36,37]. These findings in *C. glabrata* were supported in a later study in which the interaction between CgMed15A and CgPdr1 was blocked with a small molecule inhibitor, leading to a reduction in fluconazole induced expression of *CgCDR1* and *CgCDR2*, and intrinsic levels of azole resistance [38]. Another subunit, CgMed2, which is part of the tail module, was also shown to affect CgPdr1-directed expression of *CgCDR1* and *CgCDR2*, although not to the same extent as CgMed15A [32]. A more recent study in *C. glabrata* showed that a mutant lacking *CgPDR1* and *CgMED15A* was still able to maintain a plasmid containing the hyperactive *PDR1* allele as its only form of Pdr1 and still effectively drive azole resistance [39]. More recently CgPdr1-dependent transactivation was found to be mediated by a complex network of transcriptional coactivators interacting with the extreme C-terminal part of CgPdr1. These coactivators included, but were not limited to, CgMed15A [40].

Studies on *C. albicans* have also suggested that interactions between the transcription factor CaTac1 and Mediator induce azole resistance [34]. When CaTac1 was hyperactivated by xenobiotics or gain of function mutations, Mediator was recruited to the promoter of *CaCDR1* whose expression was induced, leading to increased drug efflux and azole resistance. However, in addition, this induction was equally dependent on another subunit of the tail module, CaMed3 [34]. A subunit of the kinase module of Mediator CaSSN3 was also shown to be involved in azole resistance as deletion of the gene caused decreased CaTac1-mediated resistance [33].

Mediator has also been implicated in the interaction between the transcription factor CaAce2 and the *CaERG* genes responsible for sterol biosynthesis in *C. albicans* [24,26,44]. CaAce2 is a transcription factor that regulates the expression of genes involved in a variety of cellular processes, including hyphal development, nutrient metabolism and cellular respiration [44]. Variation in *CaERG* gene expression was observed in a *∆ace2* mutant, although these changes were dependant on the cellular morphology of the fungus. When hyphal formation was induced in the *∆ace2* mutant using serum, the expression of the genes encoding the sterol biosynthetic enzymes Erg1, Erg5, Erg11 and Erg251 was decreased, however, in contrast, when grown as yeast cells, the expression of the genes encoding Erg2, Erg9, Erg10 and Erg24 increased [44]. A later study provided evidence for a role for Mediator in the expression of Ace2-regulated genes in *C. albicans* [24]. In this study, gene set enrichment analysis (GSEA) revealed similarities in the transcriptomic profile between a *∆ace2* mutant and a *∆med3* mutant, suggesting that the Med3 tail module subunit may play a role in Ace2-mediated transcriptional control. Additionally, analysis of the *∆med31* mutant displayed phenotypes consistent with altered cell membrane composition and cell wall integrity, which resulted in increased sensitivity to the polyene drug nystatin [24]. These findings reflected a study in the model yeast species *Schizosaccharomyces pombe* which described evidence for the involvement of the Med8 Mediator head module subunit on Ace2 activity [45]. In addition to playing a role in the virulence of *C. albicans*, the Mediator middle module component Med7 has also been suggested to play a role in the Ace2-regulated control of ergosterol biosynthesis genes [26]. In this study, a TAP-tagged CaMed7 protein was found to interact with CaAce2, as well as CaErg6 and CaErg25. The interaction between CaMed7, CaErg6 and CaErg25 is especially interesting in the context of antifungal resistance, as variation in the expression of *ERG251* and *ERG6* has recently been linked to altered sensitivity of *Candida* species to fluconazole [46,47].

Further evidence supporting a role for Mediator in ergosterol biosynthesis comes from studies in *C. glabrata* [48,49]. The first of these studies examined the role of the CgMed3 tail module subunit of Mediator which is encoded by two gene orthologs: *CgMED3A* and *CgMED3B*. A *C. glabrata ∆med3* mutant strain showed increased expression of *HMG1, ERG10, ERG11, ERG3* and *ERG4*. These changes in expression of sterol biosynthetic genes, in combination with changes in lipid metabolism genes, resulted in dysregulation of the membrane, leading to accumulation of squalene and lanosterol, and decreases in zymosterol, fecosterol and ergosterol in the plasma membrane compared with the wild-type strain [48]. Another study from the same group also demonstrated a role for CgMed15B, which is also found in the tail module of Mediator, in maintaining lipid metabolism and ergosterol biosynthesis homeostasis [49]. A Cg*MED15B* deletion mutant displayed abnormally high expression of specific ergosterol biosynthesis genes, such as those encoding lanosterol 14-α-demethylase (*ERG11*), methylsterol monooxygenase (*ERG2*), C-5 sterol desaturase (*ERG3*), C-22 sterol desaturase (*ERG5*) and C-24 sterol reductase (*ERG4*). Interestingly this led to an increase in zymosterol in the membrane and a major decrease in the total sterol content. Transcriptomic analysis also revealed changes in lipid metabolism upon deletion of *CgMED15B,* which also suggested changes in the structure and architecture of the plasma membrane. These changes were linked to reduced membrane integrity and fluidity, which was assessed using propidium iodide staining and anisotropy measurements [49]. Taken together the results of these studies suggest that Mediator, including subunits of the tail Module, play an important role in the control of efflux pump expression and the maintenance and regulation of sterol biosynthesis in *Candida* species, thus suggesting they have a central role in the response of medically important fungal species to antifungal drugs.

## *TLO* gene family expansion in *C. albicans*

Although the Mediator complex is conserved in all eukaryotes, there are many examples of divergence in sequence and activity of Mediator components across various species. Perhaps the most extreme example of this is the *C. albicans* Mediator tail module component Med2.

**Figure 2:**
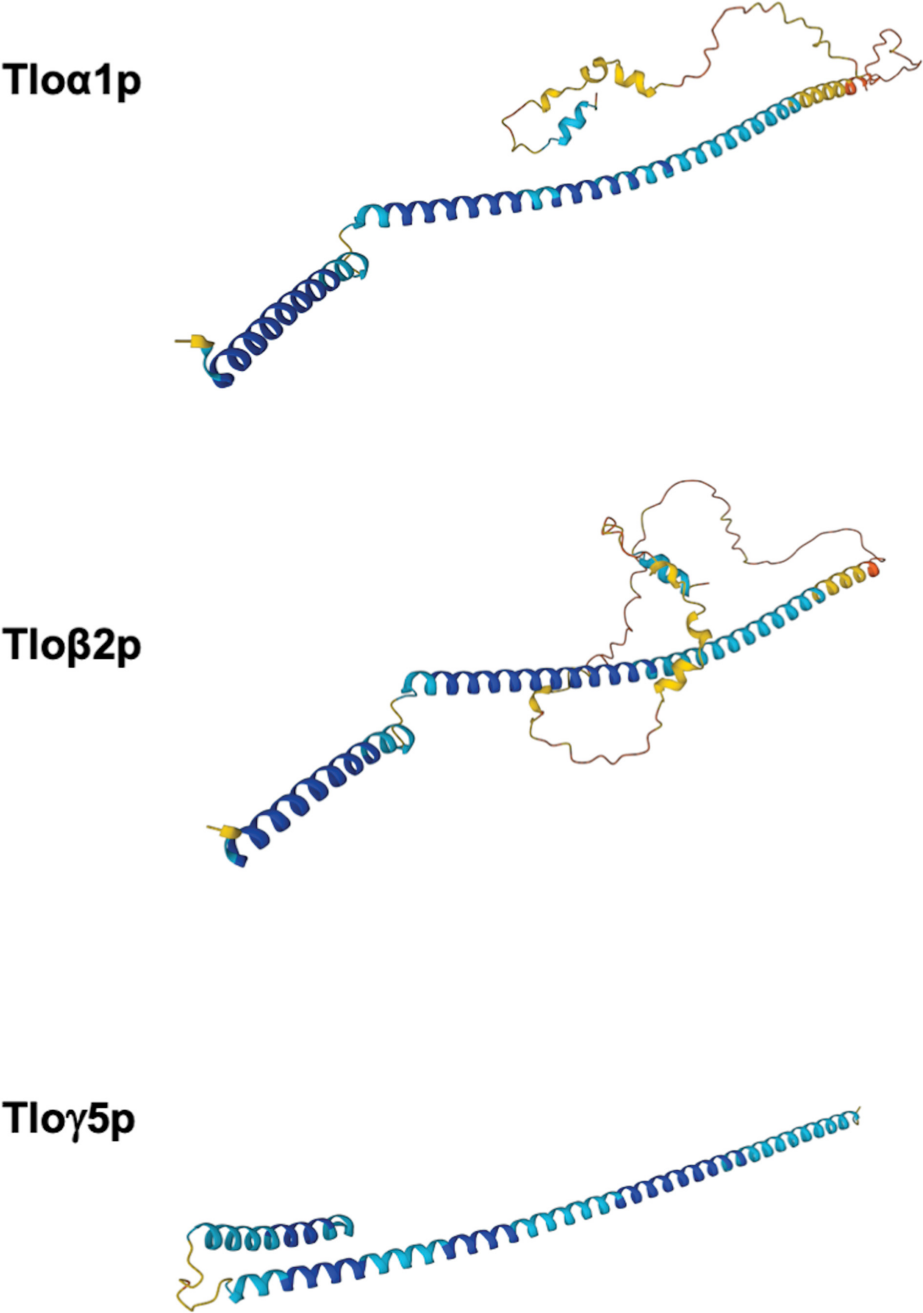
Predicted Tlo protein structures Representative structures of Tlo proteins generated by the AlphaFold structure prediction project (alphafold.ebi.ac.uk; [61,62]). The N-terminal domain of α-, β- and γ-clade Tlo proteins, encoding the Med2-like domain, has a largely α-helical structure. The C-terminal region, which has been reported to have transcriptional activating activity when fused to a DNA binding protein in one-hybrid assays, is less structured [63]. This C-terminal domain is absent in γ-clade proteins.

When the genome sequence of *C. albicans* was first described and compared with other medically important *Candida* species it was found to contain multiple paralogs of the *MED2* gene. These Med2-encoding genes were found to be located adjacent to the telomeres of almost all chromosome arms and became known as the *TLO* (telomere-associated) gene family [19]. Most *C. albicans* strains sequenced to date contain between 10 and 15 distinct *TLO* paralogs whereas other *Candida* yeast species typically encode a single *MED2* gene [50,51]. The closely related but less pathogenic species *C. dubliniensis* is unusual in that it encodes two quite divergent *MED2/TLO* genes (*CdTLO1* and *CdTLO2*) [50]. While all Tlo proteins contain a conserved Med2-like domain, phylogenetic analysis of the 14 *TLO* genes in the reference strain (SC5314) separates them into three distinct clades, referred to as α-, β- and γ-clades [52–54]. Individual clades are defined by the sequence of long terminal repeat insertions that can be found in the C-terminal domains of the Tlo proteins. In the reference strain, the largest clade is the γ-clade (*n*=7), these are also the shortest *TLO* genes at approximately 525 bp [52,53]. The γ-clade is also the only *TLO* clade that has been shown to undergo alternative splicing. The α-clade of *TLO*s (*n*=5) measure between 675 and 750 bp while the single β-clade gene *TLOβ2*, is 822 bp in length. *TLO* genes within a clade can share up to 97% similarity, and between clades the *TLO* genes are approximately 82% identical. It has been suggested that Tlo proteins display differing patterns of cellular localization, with the α- and β-clade Tlos containing a nuclear localization sequence that has been proposed to direct the proteins to the nucleus. Spliced *TLOγ* transcripts encode proteins that mainly localize to the nucleus; however, proteins from unspliced *TLOγ* transcripts have also been shown to localize to the mitochondria [52,53]. The tertiary structure of Tlo proteins predicted by the AlphaFold structure prediction tool [61,62] suggests that these proteins are largely split α helices, with the Tlo*γ* proteins lacking a less structured C-terminal region present in the Tloα and Tloβ clade proteins [63] (Figure 2). Given the large number of *TLO* genes in *C. albicans* it is not surprising that protein expression analysis has also revealed that, unlike in *C. dubliniensis*, there is a large population of ‘free’ Med2/Tlo proteins present that are not associated with the Mediator complex [19]. It is not clear what role, if any, these free Tlo proteins may have, however, artificially creating an excess of ‘free’ Tlo proteins in *C. dubliniensis* (by overexpressing *CdTLO2*, but not *CdTLO1*) increased the ability of this species to form hyphae [18].

Analysis of *TLO* gene synteny suggests that the *C. albicans TLO* gene family may have expanded from one ancestral *TLO* gene (possibly *TLOβ2*) through sub-telomeric recombination events [50]. It has been suggested, based on *in vitro* evolution experiments [55] and sequence analysis of a diverse collection of strains [54] that recombination and loss of heterozygosity can affect the copy number and clade representation of *TLOs* in *C. albicans* strains [55]. Analysis of the copy number and chromosomal positions of *TLO* genes in *C. albicans* sequenced genomes suggests that the *TLO* gene family is continuing to rapidly evolve [51].

The expansion of the *MED2/TLO* family in *C. albicans* is unique and it has been suggested that the size and diversity within this gene family could be implicated in the relative success of *C. albicans* as a successful coloniser and pathogen of humans [52,56–58]. The presence of varying pools of Mediator complexes, if individual Tlo proteins interact with different target transcription factors, could potentially facilitate rapid changes in expression of specific genes resulting in transcriptional plasticity which could enhance the ability of this organism to adapt to and colonize and cause infections in a diverse range of host anatomic niches. Evidence to support the hypothesis that Tlo proteins play a role in pathogenesis was originally obtained by investigating the role of the two *TLO* genes found in the closely related species *C. dubliniensis* (*CdTLO1* and *CdTLO2*) that share approximately 75% nucleotide identity [50]. Deletion of these two *CdTLO* genes resulted in a mutant that is defective in a range of virulence-associated traits, including the capacity to produce hyphae and to tolerate oxidative stresses [57]. In support of this, expansion of the *TLO* repertoire in wild-type *C. dubliniensis*, by heterologous expression of *C. albicans TLOβ2, TLOγ11 and TLOα12* genes, lead to an increase in virulence in the wax moth (*G. mellonella)* infection model, suggesting that increased *TLO* gene copy number (i.e., *TLO* gene family expansion) can enhance growth *in vivo* [56]. In addition, evidence for functional diversity among CaTlo proteins was obtained in experiments where clade-specific *C. albicans TLO* genes expressed in a *C. dubliniensis Δtlo1/Δtlo2* double mutant resulted in differences in the ability of specific genes/clades to reconstitute the phenotype of the wild-type parent [56]. Further evidence for functional diversity in the *CaTLO* gene family was also provided by Dunn et al. [59] when they expressed individual *CaTLO* genes under the control of an inducible promoter and observed distinct, but overlapping, phenotypes.[60–63]

Confirmation of the hypothesis that expansion and subsequent evolution of functional diversity within the expanded *C. albicans TLO* gene family has played a role in the development of *C. albicans* as a successful coloniser and cause of disease in humans has been hampered by the challenges presented in deleting all members of such a large gene family. However, the application of CRISPR-Cas9 technology to delete the entire complement of *TLO* genes in *C. albicans* offers a unique opportunity to determine the role of individual *TLO* genes and determine once and for all the role of free and Mediator-associated CaTlos in the ability of this species to cause disease and respond to antifungal agents.

## Summary

- Mediator plays an important role in the control of virulence factors expressed by human pathogenic fungi.
- Mediator is also implicated in how these fungi respond to antifungal drugs.
- Expansion of the *C. albicans TLO/MED2* gene family may play a role in the ability of this species to colonize and cause disease in humans.

## Abbreviations

ChIP: chromatin immunoprecipitation
DRE: drug responsive element
FGR: fungal gene regulator
PIC: preinitiation complex
Pol II: polymerase II
